# *SMAD4* and *NF1* mutations as potential biomarkers for poor prognosis to cetuximab-based therapy in Chinese metastatic colorectal cancer patients

**DOI:** 10.1186/s12885-018-4298-5

**Published:** 2018-04-27

**Authors:** Zhu Mei, Yang W. Shao, Peinan Lin, Xiaomin Cai, Biao Wang, Yan Ding, Xiangyuan Ma, Xue Wu, Yewei Xia, Dongqin Zhu, Yongqian Shu, Zan Fu, Yanhong Gu

**Affiliations:** 1 Department of Oncology, The First Affiliated Hospital of Nanjing Medical University, Nanjing, Jiangsu China; 2 Department of Oncology, The Affiliated Sir Run Run Hospital of Nanjing Medical University, Nanjing, Jiangsu China; 3 Translational Medicine Research Institute, Geneseeq Technology Inc., Toronto, ON Canada; 4 School of Public Health, Nanjing Medical University, Nanjing, Jiangsu China; 5 Medical Department, Nanjing Geneseeq Technology Inc., Nanjing, Jiangsu China; 6 Department of General Surgery, The First Affiliated Hospital of Nanjing Medical University, Nanjing, Jiangsu China

**Keywords:** *SMAD4*, *NF1*, Metastatic colorectal cancer, Cetuximab, Prognosis, Next-generation sequencing

## Abstract

**Background:**

Cetuximab, an anti-EGFR monoclonal antibody, is used in combination with chemotherapy in clinic to enhance the outcome in metastatic colorectal cancer (mCRC) patients with only ~ 20% response rate. To date only activating mutations in *KRAS* and *NRAS* have been identified as poor prognosis biomarkers in cetuximab-based treatment, which makes an urgent need for identification of novel prognosis biomarkers to precisely predict patients’ response in order to maximize the benefit.

**Methods:**

In this study, we analysed the mutation profiles of 33 Chinese mCRC patients using comprehensive next-generation sequencing (NGS) targeting 416 cancer-relevant genes before cetuximab treatment. Upon receiving cetuximab-based therapy, patients were evaluated for drug response, and the progression-free survival (PFS) was monitored. The association of specific genetic alterations and cetuximab efficacy was analyzed.

**Results:**

Patients carrying *SMAD4* mutations (*SMAD4*^mut^, *n* = 8) or *NF1* mutations (*NF1*^mut^, *n* = 4) had significantly shorter PFS comparing to those carrying wildtype *SMAD4* (*SMAD4*^wt^, *n* = 25) (*P* = 0.0081) or wildtype *NF1* (*NF1*^wt^, *n* = 29) (*P* = 0.0028), respectively. None of the *SMAD4*^mut^ or *NF1*^mut^ patients showed response to cetuximab when assessed at 12-week post-treatment. Interestingly, two patients carrying both *SMAD4*^mut^ and *NF1*^mut^ showed the shortest PFS among all the patients.

**Conclusions:**

Our results demonstrated that *SMAD4* and *NF1* mutations can serve as potential biomarkers for poor prognosis to cetuximab-based therapy in Chinese mCRC patients.

**Electronic supplementary material:**

The online version of this article (10.1186/s12885-018-4298-5) contains supplementary material, which is available to authorized users.

## Background

Colorectal cancer (CRC) represents a major public health issue due to its high incidence and mortality rate. In China, it is the fifth most common cancer in 2015 and causes ~ 191,000 deaths annually [1]. Comparing to other malignant tumors, CRC has a higher intra-tumoral heterogeneity and harbours higher tumor mutation burden [2, 3]. Common driver genes for CRC include *TP53*, *APC*, *KRAS*, *NRAS*, *PIK3CA*, and *SMAD4*, etc. [4, 5].

Current treatment of unresectable metastatic CRC (mCRC) in advanced stage mainly relies on fluoropyrimidine-based chemotherapies alone or in combination with Bevacizumab according to the National Comprehensive Cancer Network Clinical Practice Guidelines in Oncology (NCCN Guidelines) in Colon Cancer [6, 7]. The efficacy of chemotherapy could be further increased via the addition of cetuximab or panitumumab, which are humanized monoclonal antibodies that target the extracellular ligand binding domain of the epidermal growth factor receptor (EGFR) [8, 9]. However, only ~ 20% of patients benefit from the cetuximab-based therapy [10]. To date, prognostic biomarkers for cetuximab treatment is limited to the activating mutations of *KRAS* and *NRAS*, which have been approved for poor response to cetuximab [11–13]. Other potential biomarkers, including mutations in *BRAF* (V600E), *PIK3CA*, *SMAD4*, *PTEN*, etc., have also been reported, albeit requiring further validation [14–17]. However, all the aforementioned biomarkers could not adequately explain the poor response rate, and thus limits patients to get maximal benefit from cetuximab-based therapy.

The aim of this study was to seek better prognosis biomarkers for cetuximab-based therapy via a comprehensive analysis of the mCRC patients’ mutation profiles using next-generation sequencing (NGS) targeting 416 cancer-relevant genes. In combination with their response to cetuximab treatment, our data show that patients carrying at least one of *SMAD4* or *NF1* mutations had a higher possibility of a poor response to EGFR blockade with a shorter progression-free survival (PFS), suggesting that *SMAD4* and *NF1* mutations may play an important role in tumor progression and might function as biomarkers for poor prognosis to cetuximab-based therapy in mCRC patients.

## Methods

### Patients

This study was approved by the ethic committee of the First Affiliated Hospital with Nanjing Medical University. From 2009 to Nov 2015, tumor specimens or plasma samples were collected at the time of diagnosis from 33 Chinese patients with mCRC with written informed consents. All patients received at least one course of cetuximab treatment in combined with FOLFIRI, FOLFOX or XELOX until progressive disease (PD) was observed. Disease progression was evaluated every 6 weeks during treatment according to the Response Evaluation Criteria In Solid Tumors (RECIST) version 1.1 [18]. PFS was calculated from the first day of the administration of cetuximab-based therapy until PD. According to the Guidance for Industry Clinical Trial Endpoints for the Approval of Cancer Drugs and Biologics, partial response (PR) and complete response (CR) are considered as response to the treatment applied [19].

### Clinical sample collection and DNA extraction

Specimen collection and preparation were performed following the standard protocols approved by the First Affiliated Hospital with Nanjing Medical University, China. A minimum of 20% tumor content was required for formalin-fixed paraffin-embedded (FFPE) specimens, from which genomic DNA was extracted using QIAamp DNA FFPE Tissue Kit (QIAGEN) following the manufacturer’s instructions. Plasma was extracted from 5 to 10 ml peripheral blood collected in EDTA-coated tubes within 2 h from the blood withdrawn. Circulating tumor DNA (ctDNA) was extracted from the plasma using QIAamp Circulating Nucleic Acid Kit (QIAGEN). Genomic DNA of the whole blood was extracted with DNeasy Blood & Tissue Kit (QIAGEN) as germline control. The DNA quantity was measured on Qubit 3.0 with dsDNA HS Assay Kit (Life Technologies).

### Library preparation and sequencing

Sequencing libraries were prepared with KAPA Hyper Prep Kit (KAPA Biosystems) as per manufacturer’s instructions with optimized protocols as previously described [20]. Briefly, fragmented DNA was subjected to end-repairing, A-tailing, indexed-adapter ligation, size selection, and PCR amplification. For targeted enrichment, indexed DNA libraries were pooled together for hybridization with customized xGen lockdown probes (Integrated DNA Technologies) for 416 predefined cancer-relevant genes. Enriched libraries were amplified and subjected for NGS on Illumina Hiseq4000 platforms (Illumina) to a targeted mean coverage depth of 500× for FFPE samples or 3000× for ctDNA samples.

### Data processing

After demultiplexing, FASTQ files were processed with Trimmomatic [21] for quality control. Reads were then mapped to human reference genome 19 (hg19) using Burrows-Wheeler Aligner (BWA) [22]. Local realignment and base quality score recalibration were conducted with Genome Analysis Toolkit (GATK) [23]. Mapping rate for each sample is over 99.5% with 82% on-target rate and 99.2% of uniformity (percentage of bases over 0.2× mean coverage depth). SNV and indel mutation calling was performed using VarScan (https://dkoboldt.github.io/varscan) (< 10% minor allele frequency [MAF]) and GATK (> 10% MAF). Identified Mutations were first filtered with dbSNP and 1000 Genome data sets to remove common SNPs. Mutations identified within the whole blood controls were subtracted to exclude germline mutations where applicable. Structural variants were detected using FACTERA with default parameters [24]. ADTEx (https://adtex.sourceforge.net) was used to identify copy number variations (CNVs) with default parameters. All the genetic alterations identified were manually reviewed on Integrative Genomics Viewer (IGV) software for curation [25].

### Statistical analysis

Cox proportional hazards model was applied to assess if patients’ baseline characteristics show significant association to the PFS. To each gene where applicable, Wilcoxon ranked sum test was applied to compare the PFS of patients with or without mutations in the specified gene. Gehan-Breslow-Wilcoxon method was used to further assess the PFS curves of different patient groups based on their genotype. *P* value less than 0.05 was considered statistically significant.

## Results

### Mutation profiling in Chinese mCRC patients

**Fig. 1:**
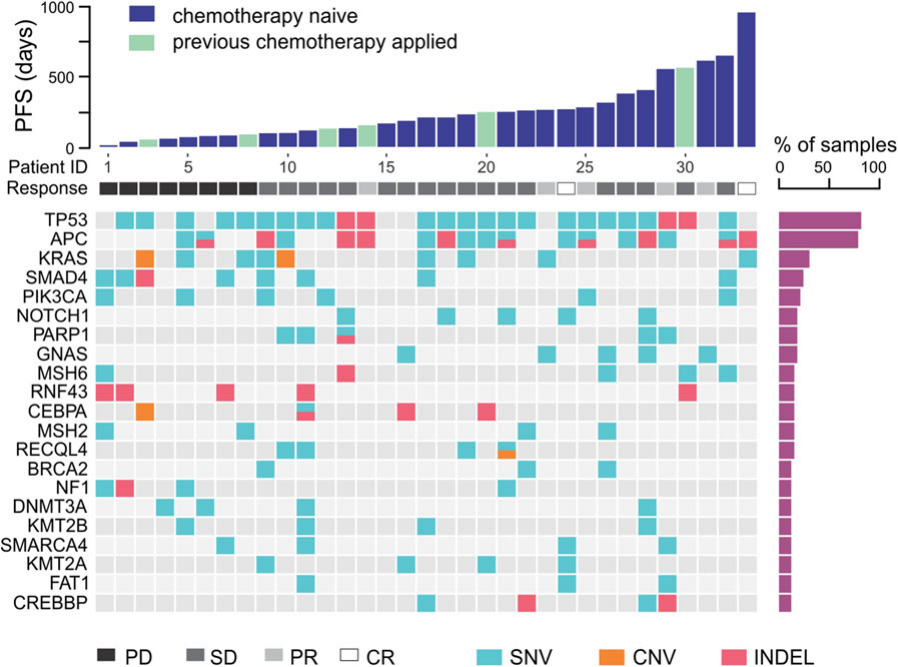
Genetic alterations detected from 33 Chinese mCRC patients using targeted NGS with a 416 cancer-related gene panel. Twenty one genes with at least 4 recurrences are shown in the figure. PFS upon receiving cetuximab are shown on the top. Different mutation types are colour coded: cyan represents single nucleotide variant (SNV), orange represents copy number variation (CNV), and red represents insert/deletion (Indel). Patients received previous chemotherapy are labeled differently as indicated in their PFS

All patients enrolled in this study were pre-screened as *KRAS* G12 and G13 mutation-negative using amplification-refractory mutation system (ARMS) [26] analysis based on the NCCN guideline that patients with mutations in *KRAS* are not suggested for cetuximab-based therapy [6]. The clinical information of the 33 Chinese mCRC patients enrolled in this study was summarized in Table 1 and the detailed clinical characteristics of each patient were listed in Additional file 1: Table S1. Pre-cetuximab-based treatment, tumor samples from 31 patients and plasma samples from 2 patients (Patient 4 and Patient 33) were analyzed using NGS targeting 416 cancer-relevant genes [20]. All patients received at least one course of cetuximab-based therapy until PD was observed. Drug response was evaluated at week 12 post-treatment, and PFS was calculated from the first day of the treatment until PD (Fig. 1). Other than the ECOG score (*P* = 0.007), no baseline characteristics are correlated to PFS according to Cox proportional hazards model (Table 1).

**Table 1:** Clinical information of patients enrolled in this study

	Patient number (percentage of patients)	*P* value*
Total patient number	33	
Age(years)		0.50
< 65	26(78.8)	
≥ 65	7 (21.2)	
Gender		0.47
Male	24(72.7)	
Female	9(27.2)	
Location of primary tumor		0.76
Colon	24(72.7)	
Rectum	9(27.2)	
Number of metastatic sites		0.15
< 3	26(84.8)	
≥ 3	7(15.2)	
Liver metastases		0.80
Yes	19(54.5)	
No	14(45.5)	
ECOG performance status		0.007**
0 or 1	24(72.7)	
2	9(27.2)	
Histological type		0.30
Adenocarcinoma	28(84.8)	
Mucinous	5(15.2)	
Previous chemotherapy treatment		0.70
No	27 (81.8)	
Yes	6 (18.2)	

*P value was calculated according to Cox proportional hazards model between PFS and the corresponding clinical information listed in the table

**Significance was found between PFS and the ECOG performance

A total number of 411 somatic alterations including single nucleotide variations (SNVs) (*n* = 320), CNVs (*n* = 38), and insertion/deletions (indels) (*n* = 53) distributed in 190 genes were detected in these patients (Fig. 1 and Additional file 2: Table S2). The most frequently mutated gene was *TP53* (75.8%), followed by *APC* (54.5%), *SMAD4* (24.2%), and *PIK3CA* (21.2%). Compared to the published results from The Cancer Genome Atlas (TCGA) [4], the mutation frequency of *TP53* was higher in our Chinese mCRC cohort (58.6% in TCGA), while only one *FBXW7* mutation (3%) was found in our data set (Additional file 2: Table S2) versus 11.4% in TCGA [4]. Surprisingly, although the 33 patients were pre-screened as *KRAS* G12 and G13 mutation-negative by ARMS method, we were able to identify 9 out the 33 patients with *KRAS* alterations (27.3%) using NGS, including exon 2 (G12 V, G12D and G13C), exon 3 (Q61R), and exon 4 (K117 and A146) mutations, as well as 2 cases with *KRAS* amplifications (Additional file 3: Figure S1 and Additional file 2: Table S2).

### Identification of *SMAD4* and *NF1* mutations as biomarkers for poor prognosis upon cetuximab-based therapy

**Fig. 2:**
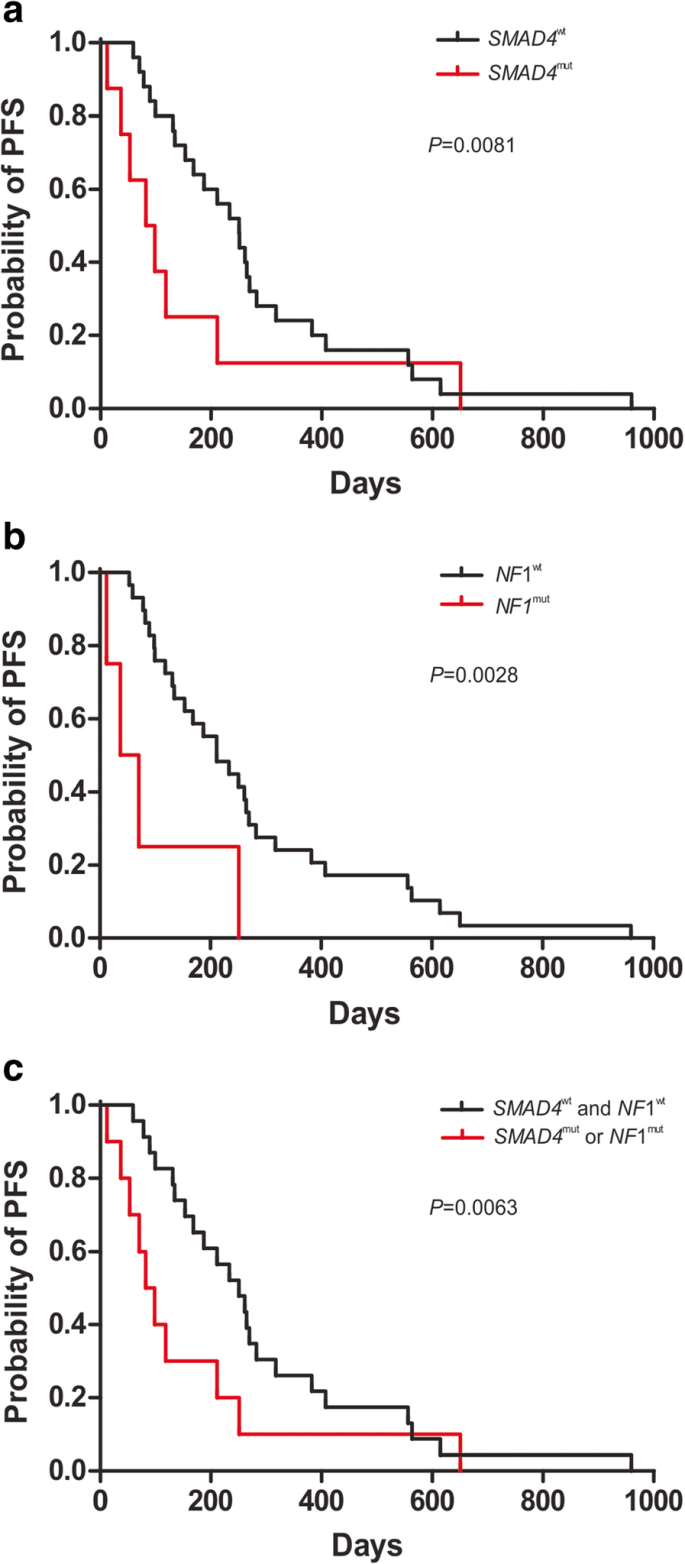
Comparison of PFS curves according to the mutation status of a defined gene or gene combination upon receiving cetuximab-based therapy. **a**. Comparison of PFS curves of the *SMAD4*^mut^ group and the *SMAD4*^wt^ group. **b**. Comparison of PFS curves of the *NF1*^mut^ group and the *NF1*^wt^ group. **c**. Comparison of PFS curves of the *SMAD4*^mut^ or *NF1*^mut^ group comparing with the *SMAD4*^wt^ and *NF1*^wt^ group. The *P* value is calculated according to Gehan-Breslow-Wilcoxon test. Figures are made using GraphPad Prism 5

As shown in Fig. 1, the PFS of all the patients range from 12 days to 959 days, with the median of 211 days. To further identify potential prognosis biomarkers for cetuximab-based therapy, we performed Wilcoxon ranked sum test [27] by comparing the PFS of patients divided by the mutation status in a specific gene. Of the 21 top mutated genes in Fig. 1, significant difference of PFS from the mutated group and the corresponding wildtype group was discovered to be caused by two genes, *SMAD4* (*P* = 0.026) and *NF1* (*P* = 0.034) (Additional file 4: Table S3). The median PFS in the *SMAD4* mutated (*SMAD4*^mut^) or *NF1* mutated (*NF1*^mut^) subsets was 90 days and 53.5 days compared to the 250 days and 211 days in the corresponding wildtype group, respectively (Table 2). Of note, two patients carrying both *SMAD4* and *NF1* mutations had the shortest PFS in our data set (12 and 37 days, respectively, Fig. 1). Furthermore, significance was also found in PFS curves between *SMAD4*^mut^ and *SMAD4*^wt^ groups (*P* = 0.0081) (Fig. 2a), and that of *NF1*^mut^ and *NF1*^wt^ groups (*P* = 0.0028) (Fig. 2b). In addition, when combining patients carrying mutated *SMAD4* and/or *NF1* (*SMAD4*^mut^ or *NF1*^mut^), the difference was still significant comparing to patients carrying both wildtype *SMAD4* and *NF1* (*SMAD4*^wt^ and *NF1*^wt^) (*P* = 0.0063) (Fig. 2c), suggesting that at least one mutated *SMAD4* or *NF1* could serve as potential biomarker to predict poor cetuximab prognosis. Furthermore, according to the drug response evaluation at week 12 post-ceuximab-based treatment, all the *SMAD4*^mut^ or *NF1*^mut^ patients showed PD or stable disease (SD), which is considered as no response according to the Guidance for Industry Clinical Trial Endpoints for the Approval of Cancer Drugs and Biologics (Fig. 1) [19].

**Table 2:** Comparison of different patient groups according to their genotype respect to PFS and drug response

	Number of patients (n=)	Median PFS (days)
Total patients	33	211
*SMAD4* ^mut^	8	90
*SMAD4* ^wt^	25	250
*NF1* ^mut^	4	53.5
*NF1* ^wt^	29	211
*SMAD4*^mut^ or *NF1*^mut^	10	90
*SMAD4*^wt^ and NF1^wt^	23	250
*KRAS* ^mut^	9	99
*KRAS* ^wt^	24	230.5

However, no difference in PFS was observed in the *KRAS*^mut^ group comparing to the *KRAS*^wt^ group in this study (Additional file 3: Figure S1). Of note, the two patients with *KRAS* amplification showed a trend of shorter PFS (53 days and 99 days, respectively) with no response to EGFR blockade, in accordance with the results described by a previous study [28].

In summary, analysis of the mutation profiles in the 33 mCRC patients in combination with the efficacy of their cetuximab-based therapy demonstrated that *SMAD4* or *NF1* mutations are promising potential biomarkers for cetuximab-based therapy in Chinese mCRC patients.

## Discussion

In this study, we analyzed the mutation landscape of 33 Chinese mCRC specimens using NGS targeting 416 cancer-relevant genes. Most frequently mutated genes are *TP53* (75.8%), *APC* (54.5%), *SMAD4* (24.2%), and *PIK3CA* (18.2%). In comparison with data from TCGA [4], *TP53* was mutated more frequently in the Chinese mCRC patients, while the mutation frequency of *FBXW7* was much lower. Furthermore, we have identified *SMAD4* and *NF1* as candidates for potential prognosis biomarkers for cetuximab-based therapy, as patients carrying mutated *SMAD4* and/or *NF1* genes had significantly shorter PFS than the *SMAD4*^wt^ and *NF1*^wt^ group.

*SMAD4* plays an important role as a common mediator in the transcriptional regulator complex in the TGF-β pathway [29], which is a bypass signaling pathway of EGFR-mediated signaling pathway for cell proliferation, differentiation and survival. It has been shown that loss of SMAD4 expression is associated to poor overall survival and poor prognosis of chemotherapy in CRC patients [30, 31]. Furthermore, studies of *SMAD4* as potential biomarkers for cetuximab prognosis have been reported previously, although insufficient and controversial. Two groups conclude that alterations in *SMAD4* are associated with a poor cetuximab prognosis [17, 32], while another study made the opposite conclusion that *SMAD4* is a biomarker for superior cetuximab prognosis [33]. Our results are in agreement with the former two studies that *SMAD4* is likely correlated to primary resistance to anti-EGFR therapy.

*NF1* encodes neurofibromin 1, which functions as a negative regulator of the RAS signaling pathway downstream of EGFR [34]. To our knowledge, our study is the first report of *NF1* as a biomarker for the prognosis of cetuximab-based therapy in mCRC. It is likely that deregulation of the TGF-β and RAS pathways due to mutations in *SMAD4* and *NF1* results in primary resistance to anti-EGFR blockade, possibly via the activation of the bypass or downstream signalling pathways of EGFR.

Although these patients were screened as *KRAS* G12 and G13 mutation-negative by ARMS method, we identified 9 out of 33 patients with *KRAS* alterations using NGS. By comparing the *KRAS*^mut^ and *KRAS*^wt^ patients, no difference was seen in PFS curves (*P* = 0.2826), which is not consistent with the current treatment guideline that patients carrying *KRAS* mutations, especially exon 2 mutations, present primary-resistance to anti-EGFR blockade [6]. The reason for the inconsistency might be due to the relative smaller sample size in this study. In addition, it has been reported in several studies that patients harbouring *KRAS* G13D mutation, comparing to other exon 2 mutations, might benefit from cetuximab-based therapy [35–37]. Mutations in other exons have different effects on cetuximab efficacy. Studies show that *KRAS* Q61 mutation has adverse effect on cetuximab prognosis while A146 seems to have little effect [16]. In our patient cohort, the *KRAS* mutations were evenly distributed in three exons at 6 spots (Additional file 3: Figure S1) after pre-screening for KRAS exon 2 mutation-negative, which might put bias on patient selection and thus makes the difference of PFS curves of *KRAS*^mut^ and *KRAS*^wt^ not significant.

## Conclusion

In this study, we have depicted pan-cancer mutation profiles from 33 Chinese mCRC patients. We further identified *SMAD4* and *NF1* mutations as potential biomarkers for poor prognosis of cetuximab-based therapy, which needs to be further validated in a larger patient cohort.

## Additional files

Additional file 1: **Table S1.** Clinical characteristics of the 33 Chinese mCRC patients enrolled in this study. (XLSX 12 kb)

Additional file 2: **Table S2.** Genetic alterations detected in targeted NGS with a 416 pan-cancer gene panel from the 33 Chinese mCRC patients. (XLSX 53 kb)

Additional file 3: **Figure S1.** Prognosis of cetuximab-based treatment between *KRAS*^mut^ and *KRAS*^wt^ patients. A. Mutation site and type of the 9 *KRAS* mutations or genetic alterations detected in this study associated with PFS and drug response to cetuximab-based therapy. *KRAS*^wt^ indicates the median PFS of wildtype *KRAS* patients (230.5 days). The minor allele frequency (MAF) of each *KRAS* mutation is displayed in red. *SMAD4* and *NF1* mutations are shown in yellow in the Fig. B. PFS curves of patients with (*KRAS*^mut^) and without (*KRAS*^wt^) mutations in *KRAS*. *P* value is calculated according to Gehan-Breslow-Wilcoxon test. Figure is made using GraphPad Prism 5. (JPEG 983 kb)

Additional file 4: **Table S3.** Statistical difference of PFS of patients with or without mutations in a specific gene. (XLSX 10 kb)

## Abbreviations

ARMS: Amplification refractory mutation system
CNV: Copy number variation
CR: Complete response
ctDNA: Circulating tumor DNA
EGFR: Epidermal growth factor receptor
FFPE: Formalin-fixed paraffin-embedded
GATK: Genome Analysis Toolkit
hg19: Human reference genome 19
IGV: Integrative Genomics Viewer
indel: Insertion/deletion
MAF: Minor allele frequency
mCRC: Metastatic colorectal cancer
NCCN: National Comprehensive Cancer Network
NGS: Next-generation sequencing
PD: Progressive disease
PFS: Progression-free survival
PR: Partial response
RECIST: Response Evaluation Criteria In Solid Tumors
SD: Stable disease
SNV: Single nucleotide variation
TCGA: The Cancer Genome Atlas
