# Supplementary figures and images for: SMAD4 and NF1 mutations as potential biomarkers for poor prognosis to cetuximab-based therapy in Chinese metastatic colorectal cancer patients
Source: BMC Cancer. 2018 Apr 27;18:479. doi: 10.1186/s12885-018-4298-5 (PMC5921972; doi:10.1186/s12885-018-4298-5)

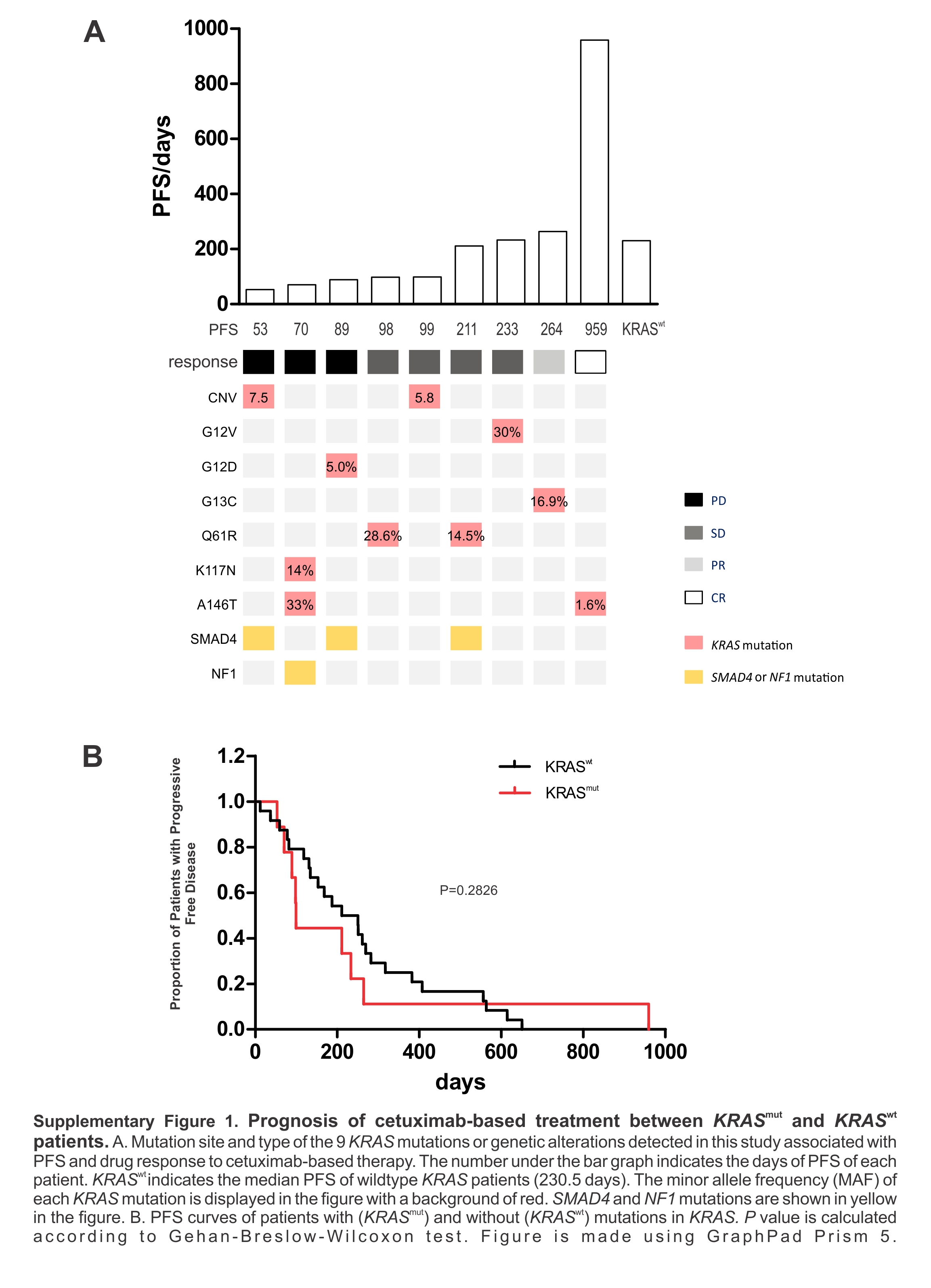

Supplement: Supplementary file 3 — Figure S1. Prognosis of cetuximab-based treatment between KRASmut and KRASwt patients. A. Mutation site and type of the 9 KRAS mutations or genetic alterations detected in this study associated with PFS and drug response to cetuximab-based therapy. KRASwt indicates the median PFS of wildtype KRAS patients (230.5 days). The minor allele frequency (MAF) of each KRAS mutation is displayed in red. SMAD4 and NF1 mutations are shown in yellow in the Fig. B. PFS curves of patients with (KRASmut) and without (KRASwt) mutations in KRAS. P value is calculated according to Gehan-Breslow-Wilcoxon test. Figure is made using GraphPad Prism 5. (JPEG 983 kb) [file 12885_2018_4298_MOESM3_ESM.jpg]
